# Chemoradiotherapy Is Associated With Improved Overall Survival in Intestinal-Type Gastric Cancer: A Retrospective Analysis

**DOI:** 10.14740/wjon2797

**Published:** 2026-07-30

**Authors:** Wei Zhang, Yu Jing He, Yi Ni Zhang

**Affiliations:** a Department of Colorectal Surgery, Sir Run Run Shaw Hospital, School of Medicine, Zhejiang University, Hangzhou 310016, Zhejiang, China; b Key Laboratory of Biological Treatment of Zhejiang Province, Hangzhou 310016, Zhejiang, China; c Nursing Department, Sir Run Run Shaw Hospital, School of Medicine, Zhejiang University, Hangzhou 310016, Zhejiang, China; d These authors contributed equally to this article.

**Keywords:** Intestinal gastric cancer, Survival, Chemoradiotherapy

## Abstract

**Background:**

Gastric cancer (GC) is a leading cause of cancer-related deaths globally, and intestinal GC accounts for more than 50% of GC cases. However, there are no studies on chemoradiotherapy effects for intestinal GC in a large population in the last 30 years.

**Methods:**

In this research, data of patients with intestinal GC between 1988 and 2017 were obtained from the Surveillance, Epidemiology, and End Results (SEER) database. Disparities in the survival by decade, age, gender, race, and therapy within the considered period were analyzed by Kaplan–Meier curves. The effect of chemoradiotherapy on survival was evaluated using a multivariable Fine–Gray regression model.

**Results:**

The results showed that, in general, an upward trend in the incidence of intestinal-type GC was observed. There were certain gender and ethnic differences in the incidence and survival of intestinal GC patients. Chemotherapy or radiotherapy can significantly influence survival of patients, and the effects were significantly associated with the age and stage of patients but not with gender or race; older patients and those with distant lesions showed poor survival. Furthermore, chemotherapy combined with radiotherapy can more effectively improve patients’ survival, compared with radiotherapy or chemotherapy alone.

**Conclusions:**

Chemoradiotherapy can more effectively improve patients’ survival, compared with radiotherapy or chemotherapy alone.

## Introduction

Gastric cancer (GC) is the fifth most diagnosed cancer worldwide [1]. Since it is frequently diagnosed at the advanced stage, it is also the third leading cause of cancer-related deaths imposing a considerable health burden globally [2]. The incidence and mortality of GC have changed due to the changes in living conditions and the environment. GC is mainly categorized into two types (intestinal type and diffuse type) according to Lauren’s classification based on the glandular structure. The two GC types show distinct clinical characteristics and type-specific genetic and epigenetic alterations, facilitating the understanding of the pathogenesis of GC [3]. Intestinal GC is related to atrophic gastritis, intestinal metaplasia, and atypical hyperplasia, which is usually well-differentiated and more common in areas with a high incidence of GC. *H. pylori* seropositivity [4] and EBV infection [5, 6] are also shown to be associated with an increased risk of GC. The occurrence of intestinal GC is a multi-step process, involving the alteration of a variety of genes and the microenvironment, including oncogene activation (CTNNB1 [7, 8]), the loss of tumor suppressor genes (RUNX1 [9] and CDH1 [10, 11]), damage to chromosomal DNA [12], and immune suppression [13]. Although medicine has developed rapidly in recent years, the survival of intestinal GC patients has not significantly improved and the mechanisms underlying the pathogenesis and drug resistance are not clear yet. We aimed to analyze the clinical data of intestinal GC patients to gain basic and clinical research insights for GC.

## Materials and Methods

### Data sources

The data were obtained from the SEER program of the National Cancer Institute, USA. Patients with intestinal GC are analyzed in the SEER database according to the guidelines of the World Health Organization International Classification of Diseases for Oncology.

### Data collection

The patients were diagnosed as intestinal GC between 1988 and 2017, selected using ICD-O-3 site codes (stomach) and ICD-O-3 histology codes (8144/3). The data included the incidence and the survival states of patients with intestinal GC spanning three decades, which were obtained from the original nine SEER sites (the states of Connecticut, Iowa, New Mexico, Utah, and Hawaii and the metropolitan areas of Atlanta, Detroit, San Francisco-Oakland, and Seattle-Puget Sound). Those diagnosed by autopsy or merely reported to have GC in death certificates were excluded. The patients were categorized by sex, race (White, Black, and others), and age at diagnosis (0–49, 50–59, 60–69, and 70+ years).

In the SEER database, the treatment classification of chemotherapy, radiotherapy, and chemoradiotherapy (combination of chemotherapy and radiotherapy) was directly derived from standardized recorded variables. Due to the limitation of SEER database coding, we cannot further distinguish concurrent chemoradiotherapy from sequential chemoradiotherapy; the term “chemoradiotherapy” in this study refers to patients who received both chemotherapy and radiotherapy in any sequence.

### Research design

To assess the influence of chemoradiotherapy (including both sequential and concurrent treatment modes based on SEER database coding criteria) on survival of intestinal-type GC patients, we initially enrolled intestinal-type GC patients between 1988 and 2017. The incidence and survival trends associated with intestinal-type GC were examined. Following this, we investigated the effects of chemotherapy, radiotherapy, and combined chemotherapy with radiotherapy on the survival outcomes of intestinal-type GC patients.

In the SEER database, the treatment classification of chemotherapy, radiotherapy, and chemoradiotherapy (combination of chemotherapy and radiotherapy) was directly derived from standardized recorded variables. Due to the limitation of SEER database coding, we cannot further distinguish concurrent chemoradiotherapy from sequential chemoradiotherapy.

### Statistical analysis

Patient survival was analyzed from the date of diagnosis to the date of death. The current study was designed to identify trends in the clinical outcomes of patients over time. Kaplan–Meier curves were constructed to estimate the overall survival, and differences between the curves were assessed using the two-tailed log-rank test. Stata 12.0 (StataCorp) was used for data analysis. A P-value < 0.05 was considered statistically significant.

## Results

### Patient characteristics

**Figure 1:**
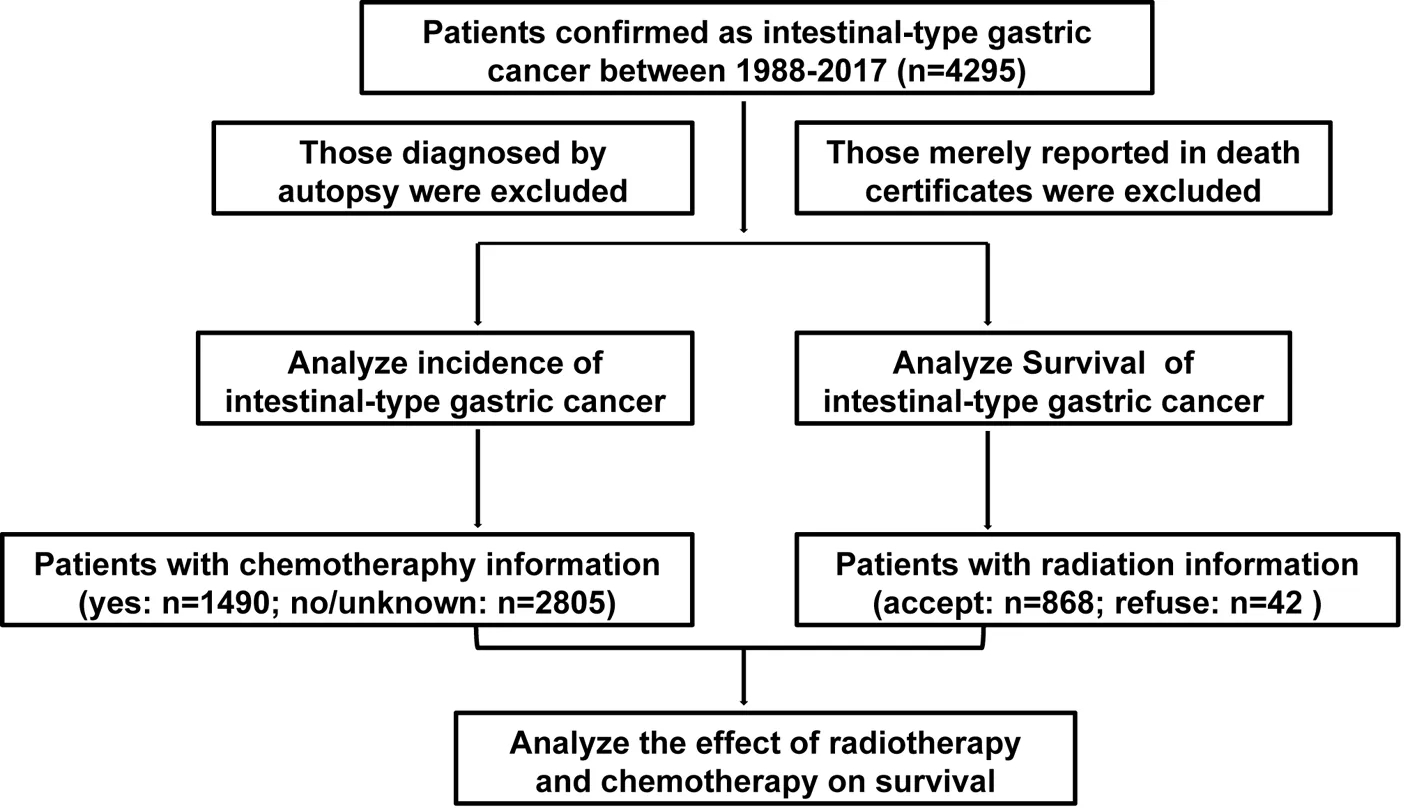
Diagrammatic sketch of the research.

The research design is shown in Figure 1. The study population consisted of 4,295 patients with intestinal GC, including 2,292 men (53.4%), 1,193 women (27.8%), and 810 gender-unknown patients (18.9%) (Table 1). There were 1,613 (37.6%) White patients, 426 (9.9%) Black patients, 1,436 (33.4%) patients of other races, and 820 (19.1%) race-unknown patients. As shown in Table 1, most of the patients with intestinal GC were over 60 years old (68%). To explore the relationship between survival of patients and therapy, we further selected 1,490 patients who received chemotherapy and 868 patients who received radiotherapy.

**Table 1:** Characteristics of Patients With Intestinal-Type GC

Characteristics	N = 4,295	Percentage (%)
Sex		
Male	2,292	53.3
Female	1,193	27.8
Unknown	810	18.9
Race		
White	1,613	37.6
Black	426	9.9
Other	1,436	33.4
Unknown	820	19.1
Age		
< 60	553	12.9
≥ 60	2,922	68.0
Unknown	820	19.1
Stage		
Distant	820	19.1
Localized	1,084	25.2
Regional	1,320	30.7
Unknown	1,071	25.0
Chemotherapy		
Yes	1,490	34.7
No/unknown	2,805	65.3
Radiotherapy		
Yes	868	20.2
Refuse	42	1.0
Unknown	3,368	78.8

GC: gastric cancer.

### Trends in the incidence of patients with intestinal GC

**Figure 2:**
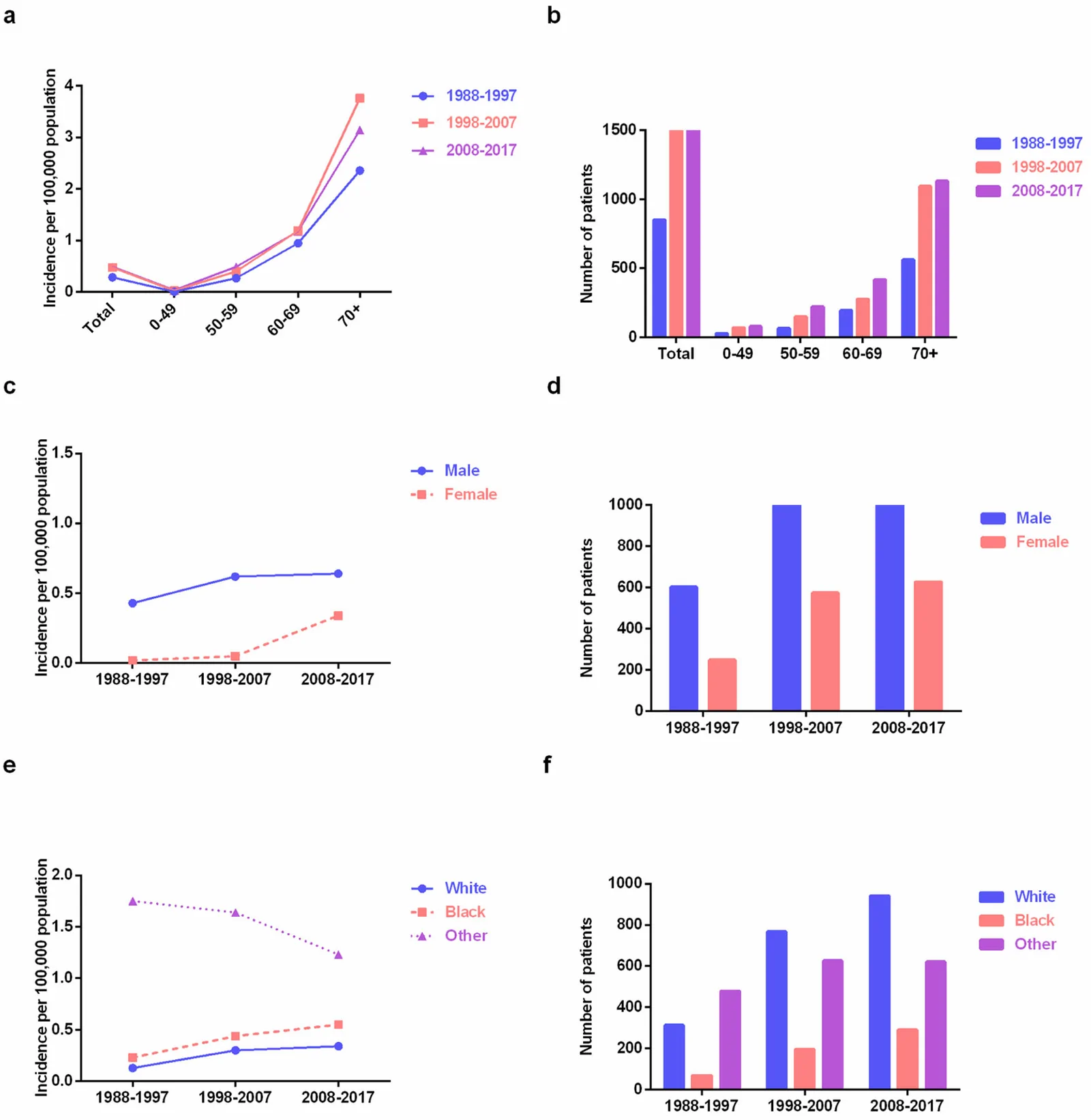
The incidence rate and the number of patients with intestinal GC by year, gender, and race (a, c, e: incidence rate per 100,000; b, d, f: number). GC: gastric cancer.

As shown in Figure 2a, b and Table 2, the number of patients showed an increasing trend, and the incidence of intestinal GC also increased continuously (the increasing trend in the third decade was not obvious and even decreased in certain age groups). Similar trends were also observed in the three decades in most age groups. While the incidence decreased in the 60–69 and 70+ groups in the third decade.

**Table 2:** The Incidence of Intestinal-Type Gastric Cancer According to Age Group and Decade Within Sex and Race Groups From 1988 to 2017 at the Nine Original SEER Sites

Variable	Age group	Year at diagnose		
		1988–1997	1998–2007	2008–2017
Total	0–85+	0.29 (852)	0.48 (1,591)	0.49 (1,852)
	0–49	0.01 (28)	0.03 (70)	0.04 (79)
	50–59	0.27 (65)	0.4 (148)	0.49 (223)
	60–69	0.95 (196)	1.19 (277)	1.18 (417)
	70+	2.36 (563)	3.76 (1,096)	3.14 (1,133)
Sex				
Male	0–85+	0.43 (603)	0.62 (1,016)	0.64 (1,224)
	0–49	0.02 (23)	0.05 (53)	0.05 (57)
	50–59	0.5 (56)	0.63 (109)	0.73 (160)
	60–69	1.55 (148)	1.74 (188)	1.84 (315)
	70+	4.03 (376)	5.59 (666)	4.36 (692)
Female	0–85+	0.17 (249)	0.34 (575)	0.34 (628)
	0–49	0 (5)	0.02 (17)	0.02 (22)
	50–59	0.05 (9)	0.19 (39)	0.25 (63)
	60–69	0.43 (48)	0.7 (89)	0.58 (102)
	70+	1.31 (187)	2.54 (430)	2.25 (441)
Race				
White	0–85+	0.13 (313)	0.3 (768)	0.34 (941)
	0–49	0.01 (13)	0.02 (35)	0.03 (47)
	50–59	0.13 (27)	0.25 (76)	0.31 (111)
	60–69	0.38 (65)	0.66 (123)	0.76 (214)
	70–79	0.98 (208)	2.19 (534)	1.94 (569)
Black	0–85+	0.23 (69)	0.44 (197)	0.55 (290)
	0–49	0.01 (3)	0.04 (14)	0.05 (14)
	50–59	0.41 (8)	0.79 (28)	0.82 (42)
	60–69	1.70 (27)	2.04 (48)	2.51 (93)
	70+	2.04 (31)	4.73 (107)	4.33 (141)
Other	0–85+	1.75 (470)	1.64 (626)	1.23 (621)
	0–49	0.06 (12)	0.09 (21)	0.06 (18)
	50–59	1.45 (30)	1.16 (44)	1.28 (70)
	60–69	5.78 (104)	4.47 (106)	2.85 (110)
	70–79	20.5 (324)	17.26 (455)	10.61 (423)

Data are incidence per 100,000 people by year of diagnosis, with the number of patients in parentheses.

Compared with females, males exhibited a markedly higher incidence per 100,000 and a higher actual count (Fig. 2c, d). Moreover, the incidence continued to increase during the three decades in females but it decreased significantly in the third decade in males (Fig. 2c).

By race, other races showed a higher incidence rate than that of White and Black races. The incidence in White and Black races showed an upward trend but it reduced in other races in the third decade (Fig. 2e). The number of White patients was also higher than the Black or other race patients in the latter two decades, while in the first decade, other races showed more cases (Fig. 2f).

### Survival estimates of intestinal-type GC patients

**Figure 3:**
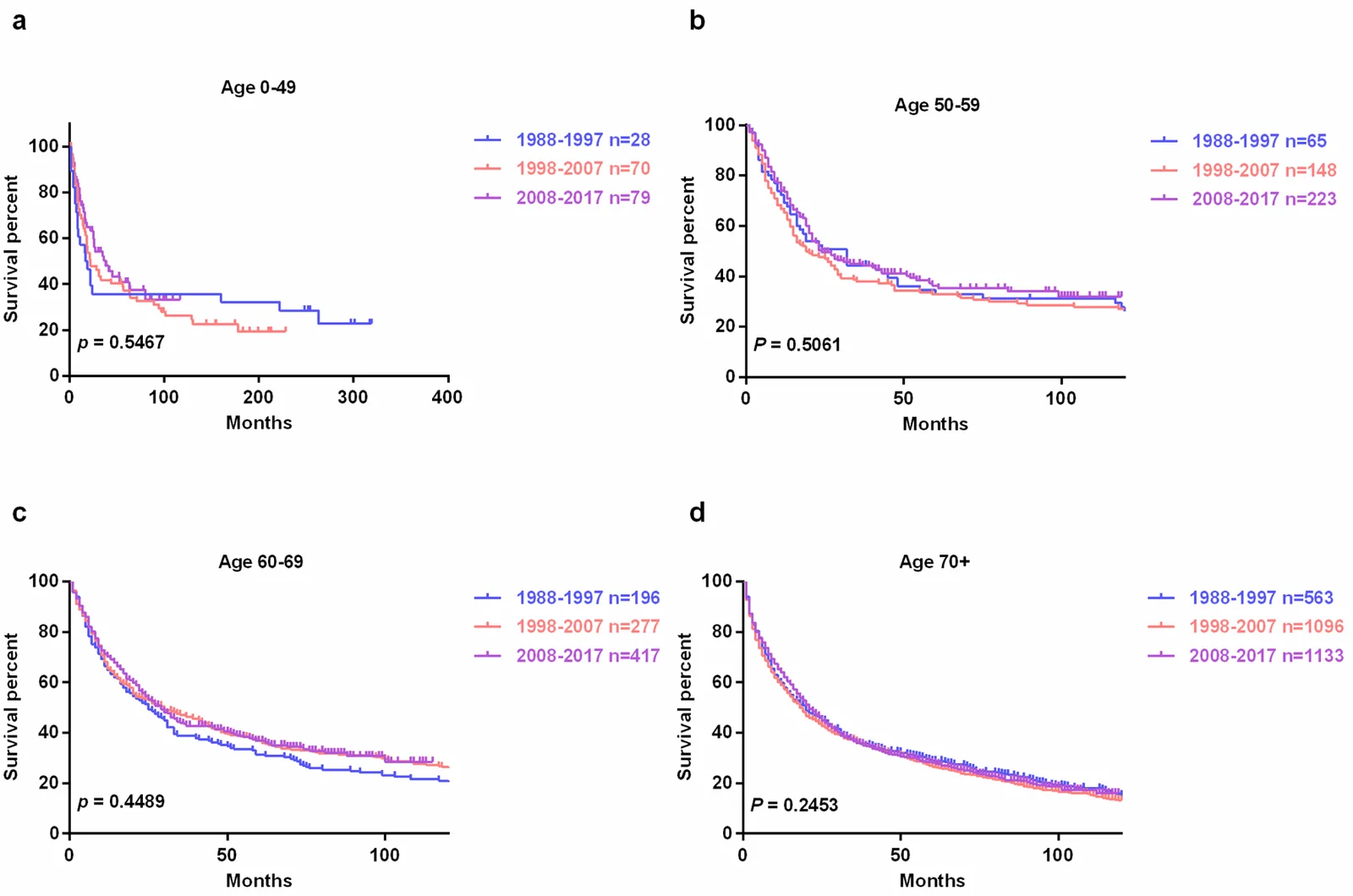
Kaplan–Meier curves of patients with intestinal GC by age during three decades. GC: gastric cancer.

**Figure 4:**
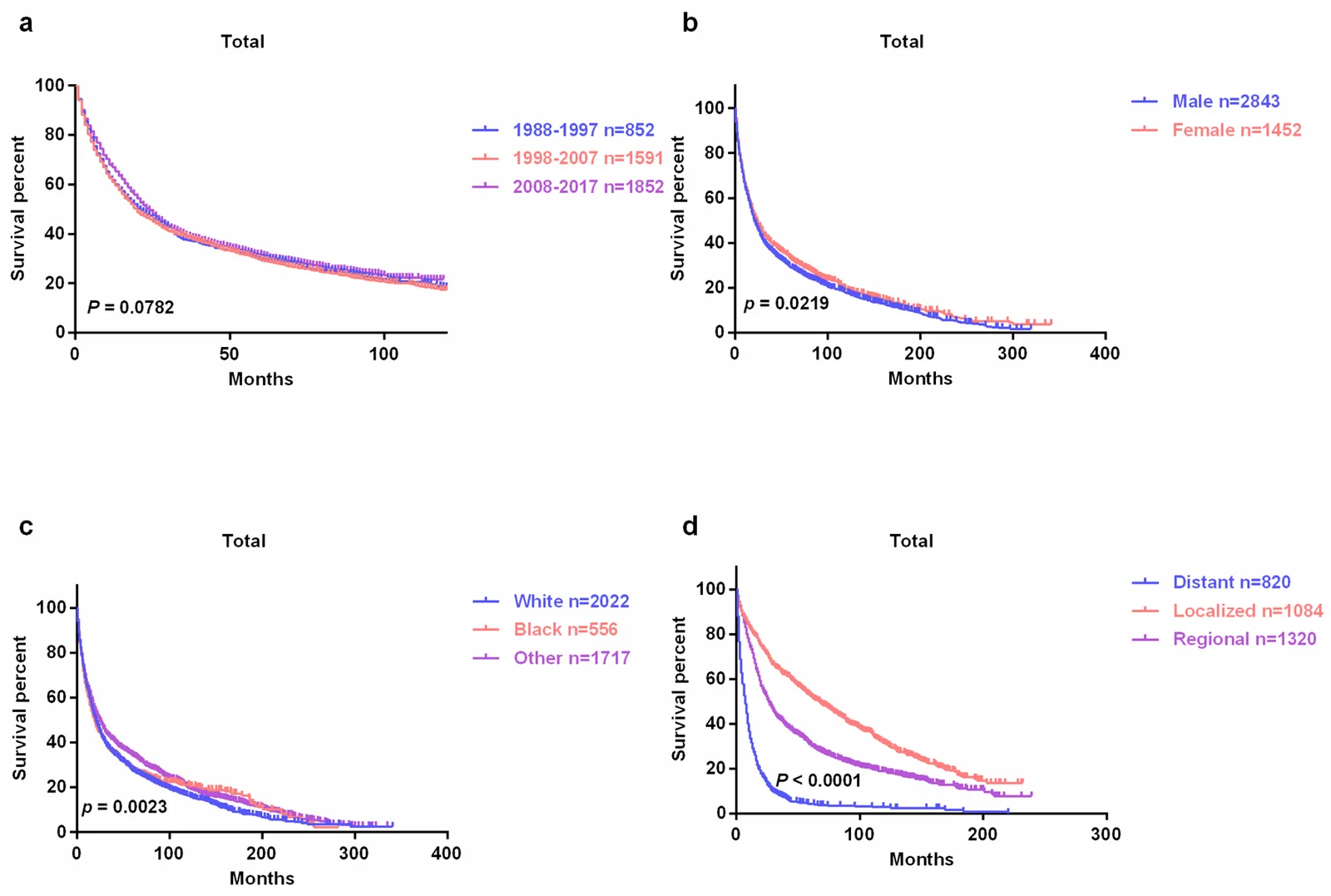
The overall trend of Kaplan–Meier curves of patients with intestinal GC during three decades (a), and the survival trends by sex (b), race (c), and stage (d). GC: gastric cancer.

The Kaplan–Meier survival analysis revealed that the overall survival rate among patients with intestinal-type GC remained stagnant from 1988 to 2017. This trend persisted across all age demographics, as depicted in Figure 3. There were certain gender, ethnic, and stage differences in the survival of intestinal GC patients: females showed a significantly longer survival time than males (P = 0.0219), the Black race showed a slightly poor prognosis than White and other races (P = 0.0023), and survival of patients with localized and regional lesion were significantly better than that of patients with distant lesion in primary disease state (P < 0.0001) (Fig. 4).

### The effect of chemoradiotherapy on the survival of intestinal GC patients

**Figure 5:**
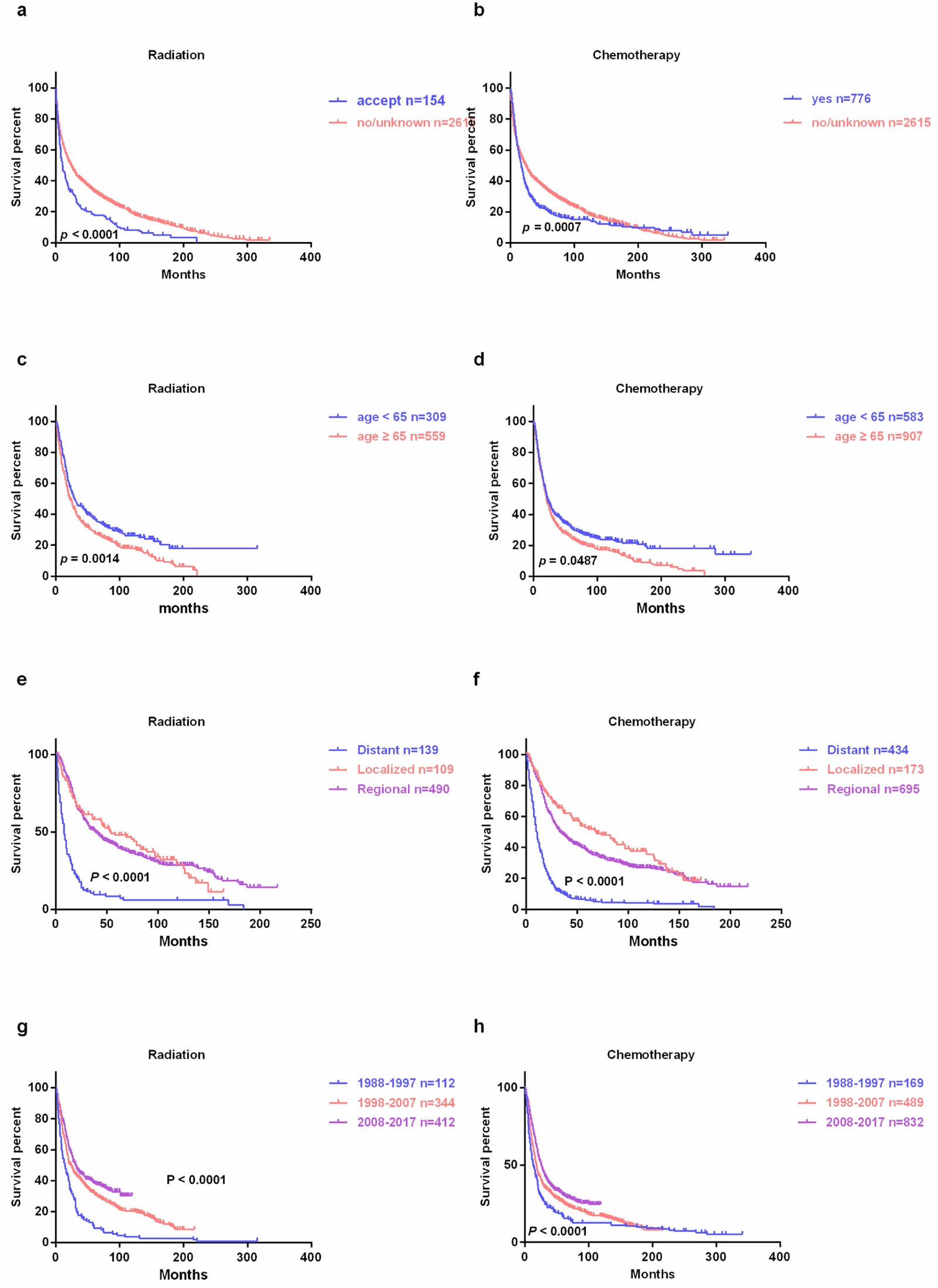
The survival of patients with chemotherapy and radiotherapy. (a, c, e, g) Survival effect of radiotherapy by the decision to accept radiotherapy, stage, age, and year. (b, d, f, h) Survival effect of chemotherapy by the decision to receive chemotherapy, stage, age, and year.

To comprehend the effect of chemoradiotherapy on intestinal GC patients, we selected 1,490 patients with complete chemotherapy information and 910 patients with integrated radiotherapy information (Fig. 1). The results in Figure 5a indicate that the survival of patients who received radiation therapy was significantly worse than those who did not (P < 0.0001); the effect of radiation therapy was associated with age and stage of patients: older patients and those with distant lesions showed poor survival (P = 0.0014 and P < 0.0001, respectively; Fig. 5c, e).

**Figure 6:**
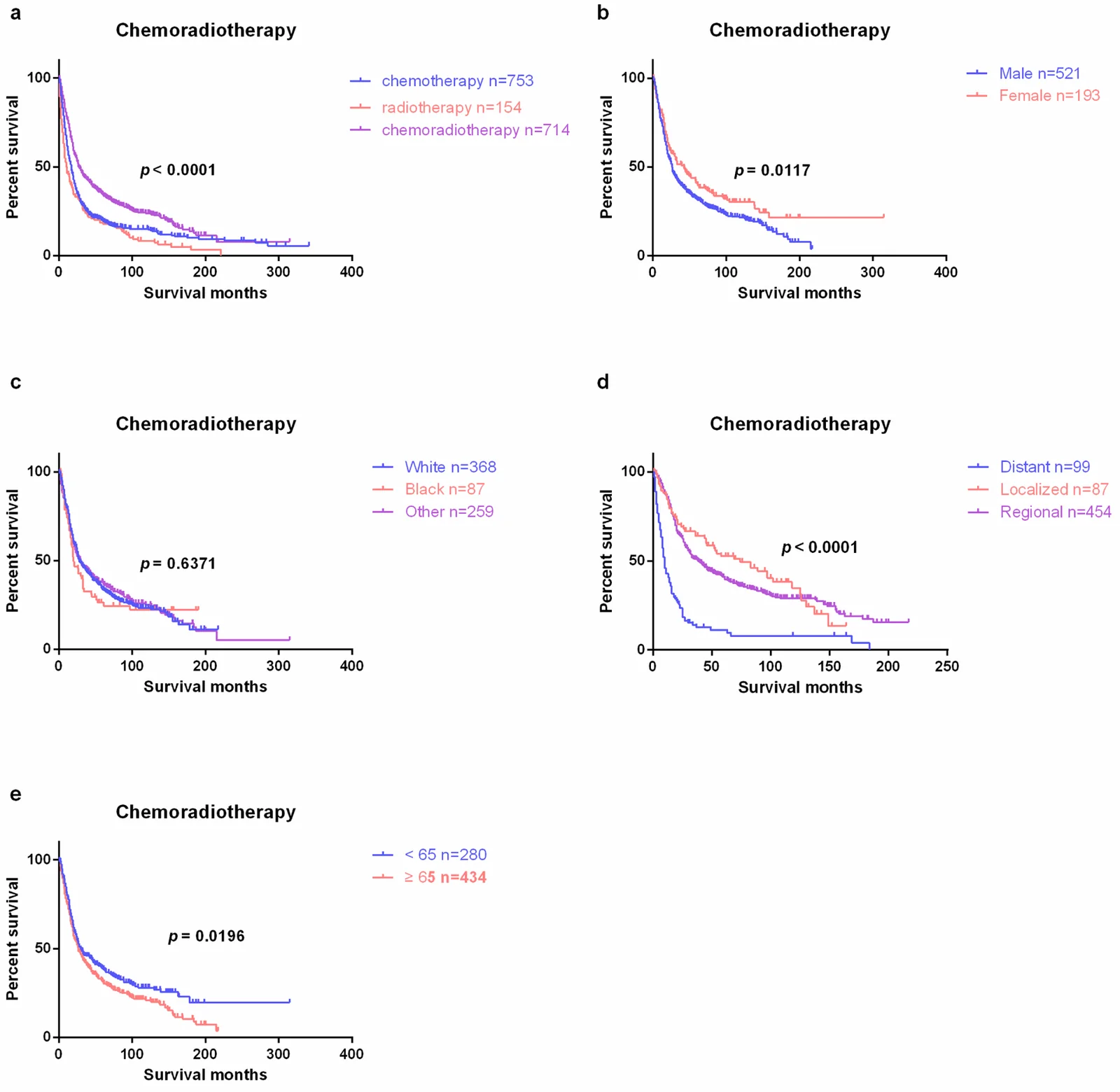
The survival of patients with chemoradiotherapy by the decision to accept chemoradiotherapy, sex, race, stage, and age.

Furthermore, Supplementary Material 1 (wjon.elmerpub.com) indicated the background characteristics in the chemotherapy yes and no/unknown groups. Similar trends were observed in the chemotherapy group (P = 0.0007, P = 0.0487, and P < 0.0001, respectively; Fig. 5b, d, and f). The chemotherapy and radiotherapy effect significantly improved over time, especially in the third decade (Fig. 5g, h). However, the effects of chemotherapy or radiotherapy were not associated with sex and race of patients (Supplementary Material 2, wjon.elmerpub.com). Furthermore, it was found that combined chemoradiotherapy yielded superior survival benefits compared with single chemotherapy or single radiotherapy (P < 0.0001), and the survival effects were related with sex (P = 0.0117), stage (P < 0.0001), and age (P = 0.0196), but not race (P = 0.6371) (Fig. 6).

**Figure 7:**
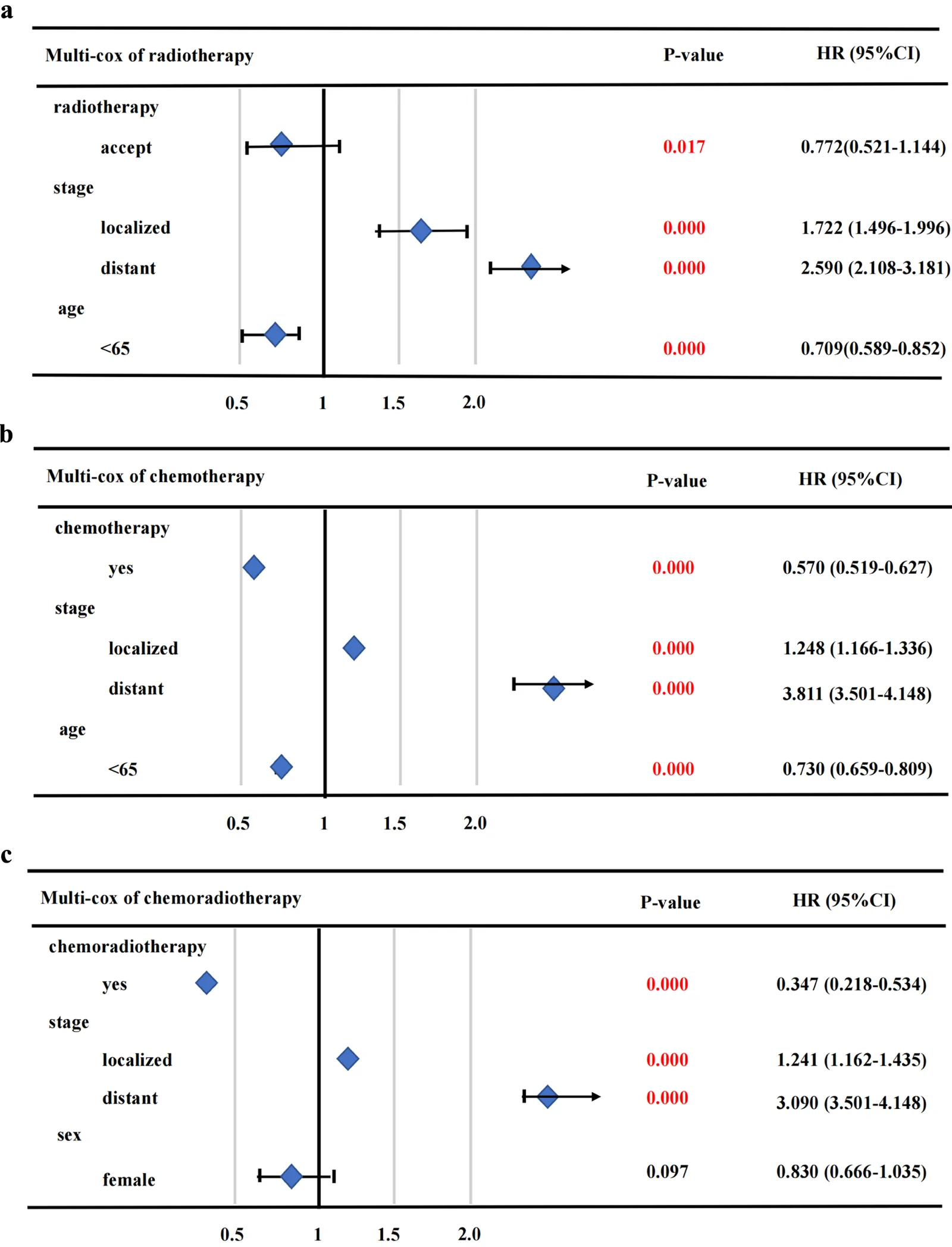
Multivariate analysis of the independent risk factors that influence the survival of patients with intestinal GC by radiotherapy (a), chemotherapy (b), and chemoradiotherapy (c).

The univariate and multivariate analyses demonstrated that the decision to accept radiotherapy and the stage and age of patients were independent risk factors affecting the prognosis of intestinal GC in the radiotherapy group (Table 3 and Fig. 7a). In the chemotherapy group, the independent risk factors affecting the prognosis of patients with intestinal GC included the decision to receive chemotherapy and stage and age of patients (Table 4 and Fig. 7b). By contrast, in chemoradiotherapy group, the decision to receive chemoradiotherapy and stage were independent risk factors affecting the prognosis of patients with intestinal GC (Table 5 and Fig. 7c).

**Table 3:** Univariate Analysis of Radiotherapy on Overall Survival

Subtype	HR (95% CI)	^P^
Sex		
Female	1	
Male	1.067 (0.8989–1.269)	0.4614
Radiotherapy		
Accept	1	
Refuse	1.368 (0.9690–2. 173)	0.0051
Age		
< 65	1	
≥ 65	1.218 (1.036–1.428)	0.0124
Stage		
Distant	1	
Localized	0.266 (0.197–0.358)	< 0.0001
Regional	0.269 (0.217–0.332)	< 0.0001
Race		
White	1	
Black	0.9865 (0.7646–1.272)	0.9155
Other	1.001 (0.8482–1. 182)	0.9895

CI: confidence interval; HR: hazard ratio.

**Table 4:** Univariate Analysis of Chemotherapy on Overall Survival

Subtype	HR (95% CI)	P
Sex		
Female	1	
Male	1.127 (0.9873–1.287)	0.0809
Chemotherapy		
Accept	1	
No/known	1.380 (1.281–1.480)	< 0.0001
Age		
< 65	1	
≥ 65	1.215 (1.077–1.378)	0.0020
Stage		
Distant	1	
Localized	0.232 (0.183–0.294)	< 0.0001
Regional	0.305 (0.264–0.352)	< 0.0001
Race		
White	1	
Black	1.026 (0.8453–1.247)	0.7928
Other	1.020 (0.8951–1. 164)	0.7638

CI: confidence interval; HR: hazard ratio.

**Table 5:** Univariate Analysis of Chemoradiotherapy on Overall Survival

Subtype	HR (95% CI)	P
Sex		
Female	1	
Male	1.299 (1.061–1.564)	0.0117
Chemoradiotherapy		
Accept	1	
No/known	1.279 (1.164–1.424)	< 0.0001
Age		
< 65	1	
≥ 65	1.219 (0.9610–1.545)	0.1063
Stage		
Distant	1	
Localized	0.278 (0.196–0.394)	< 0.0001
Regional	0.326 (0.257–0.416)	< 0.0001
Race		
White	1	
Black	1.122 (0.829–1.518)	0.455
Other	0.957 (0.777–1. 179)	0.680

CI: confidence interval; HR: hazard ratio.

## Discussion

The current research showed an upward trend in the incidence of intestinal GC. There were certain gender and ethnic differences in the incidence and survival of intestinal GC patients. Also, the survival was significantly associated with the age and stage of patients but not the gender or race by chemotherapy or radiotherapy. In addition, the survival was significantly associated with the sex, age, and stage of patients but not race by chemoradiotherapy.

Although in this study, the overall incidence of intestinal GC showed an increasing trend, there was a slight decrease in the third decade. This result may be attributed to *H. pylori* eradication [14, 15] and the use of pharmacological or natural compounds (NSAIDs) [16, 17]. The incidence in males and other races showed a significant decrease, while the incidence in females and White and Black races showed an increase. Previous research showed that the incidence of GC was higher in males and the Asian race [18], which was not consistent with our results. The study also showed that the increased speed of female and Asian race is much faster than that of males and White and Black races [18]. The differences in results may be because the previous study focused on patients in a high GC incidence area, while our study considered cases diagnosed in the USA.

Notably, the overall survival of intestinal GC patients failed to be improved over the three decades despite the rapid development of diagnostic and therapeutic technologies, which is a counterintuitive finding. Despite the popularization of gastroscopy and endoscopic ultrasound boosting early diagnosis rate, the majority of intestinal GC cases in the United States are still diagnosed at advanced clinical stages. In addition, this study included unselected population-based real-world cases covering all age groups; population aging and increased preoperative comorbidities in elderly patients further offset the survival benefits brought by minimally invasive robotic/laparoscopic surgery, modern conformal radiotherapy, and novel immune checkpoint inhibitors such as nivolumab. These confounding population-level factors collectively lead to the stagnant overall survival trend in this large retrospective cohort.

In the current study, the differences in the survival of intestinal GC patients between genders and across races were significant, which is consistent with the results of a previous study [19]. The findings of the aforementioned study are inconsistent with our own research outcomes. Two potential reasons account for this outcome: firstly, the restricted sample size within our study; secondly, the primary focus of the study on the United States, despite the prevalent high incidence of gastric cancer in Asia.

Although surgical resection is still the main treatment for GC, radiotherapy and chemotherapy also play an important role [20]. Our study showed that the survival of intestinal GC patients was significantly associated with the age and stage of patients but not gender or race. Late-stage GC patients are rarely subjected to endoscopic resection, and the tumors are usually too large to be resected directly [20]. Therefore, chemotherapy and radiotherapy may be administered.

Our study found that single radiotherapy group had worse survival outcomes than the non-radiotherapy group, which is easily misinterpreted as radiotherapy being harmful to patients. This study is a retrospective population-based analysis using the SEER database. Treatment decisions (chemotherapy/radiotherapy/chemoradiotherapy) were not randomly assigned but determined by clinical practice, patient condition, tumor stage, and attending physicians. Therefore, treatment selection bias was unavoidable. Patients who received radiotherapy or chemotherapy tended to have more advanced tumor stage, older age, or worse physical status, which may partly explain the relatively poor survival in the monotherapy group observed in this study. These baseline differences may confound the real therapeutic effect. In clinical practice, radiotherapy may be used in patients with distant metastatic disease for palliative purposes to relieve gastrointestinal obstruction, tumor bleeding, and cancer-related pain, rather than radical tumor resection. Therefore, the poorer survival in the radiotherapy group may also be related to the inclusion of some palliative-treated advanced patients.

Previous research demonstrated that patients who received chemotherapy showed longer overall survival. For instance, in the randomized phase 3 ACTS-GC trial (NCT00152217), adjuvant S-1 for 1 year indicated a survival benefit compared with surgery alone (5-year OS, 72% vs. 61%) [21]. However, the effect of radiotherapy is less certain. In the INT 0116 trial, a 9-month overall survival benefit was observed for adjuvant chemoradiation compared with curative intent-surgery in patients with gastroesophageal adenocarcinoma [22]. Our results also showed a survival benefit for chemotherapy and radiotherapy, which is consistent with the previous results. In addition, results of phase III trial in 2015 showed that both adjuvant chemotherapy and chemoradiotherapy are tolerated and equally beneficial in preventing relapse [23], and results of another research turned out that postoperative chemoradiotherapy did not improve overall survival compared with postoperative chemotherapy in patients with resectable GC treated with adequate preoperative chemotherapy and surgery [24]. However, in our research, it was found that chemotherapy combined with radiotherapy can more effectively improve patients’ survival, compared with radiotherapy or chemotherapy alone.

In conclusion, we revealed the differences in the survival of intestinal-type GC patients between gender, age, and stage by chemotherapy and radiotherapy. We also demonstrated the survival benefits of chemoradiotherapy, which further indicated that more effective basic and clinical studies are needed to improve the early diagnosis rate and treatment effects of intestinal-type GC. Meanwhile, several limitations of this study should be noted: relevant confounding factors were not adjusted via propensity score matching due to its retrospective nature, detailed tumor subsite information was unavailable in the SEER database, and we could not distinguish sequential and concurrent chemoradiotherapy based on official database coding. Accordingly, future prospective studies with complete clinical and anatomical data are required to refine individualized diagnostic and therapeutic strategies for intestinal-type GC.

## Supplementary Material

Suppl 1 Characteristics of intestinal-type GC patients by chemotherapy. Other: race including Asian or Pacific Islander and American Indian/Alaska Native.

Suppl 2 The survival of patients with chemotherapy and radiotherapy by sex (a, c) and race (b, d).

## Data Availability

The datasets generated and analyzed during the current study are obtained from SEER database.
