# Supplementary material for: Chemoradiotherapy Is Associated With Improved Overall Survival in Intestinal-Type Gastric Cancer: A Retrospective Analysis
Source: World J Oncol. 2026 Jul 30;17(5):633–45. doi: 10.14740/wjon2797 (PMC13568766; doi:10.14740/wjon2797)
Supplement: Supplementary file 1 [file wjon-17-05-633-s002.pdf]

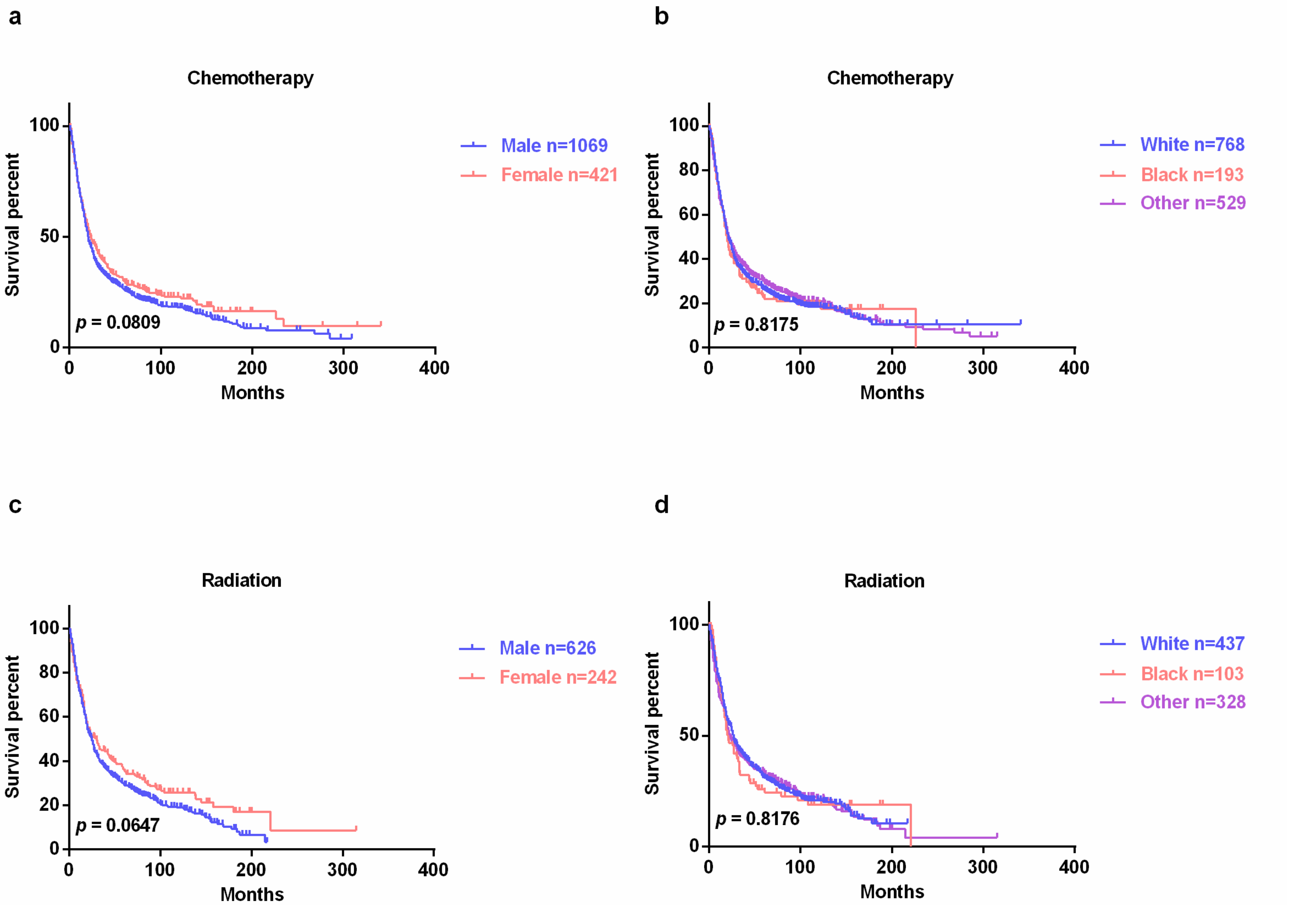
a
Chemotherapy
Survival percent
Months
Male n=1069
Female n=421
p = 0.0809
b
Chemotherapy
Survival percent
Months
White n=768
Black n=193
Other n=529
p = 0.8175
c
Radiation
Survival percent
Months
Male n=626
Female n=242
p = 0.0647
d
Radiation
Survival percent
Months
White n=437
Black n=103
Other n=328
p = 0.8176
