# Supplementary material for: Chemoradiotherapy Is Associated With Improved Overall Survival in Intestinal-Type Gastric Cancer: A Retrospective Analysis
Source: World J Oncol. 2026 Jul 30;17(5):633–45. doi: 10.14740/wjon2797 (PMC13568766; doi:10.14740/wjon2797)
Supplement: Supplementary file 2 [file wjon-17-05-633-s001.docx]

**Suppl 1.** Characteristics of intestinal-type GC patients by chemotherapy.

| **Characteristics** | **Chemotherapy** |
| --- | --- |
|  | **Yes No/Unknown** |
|  | **N(1490) percent(%) N(2805) percent(%)** |

| **Sex**  Male | 1069 | 71.7 | 1774 | 63.2 |
| --- | --- | --- | --- | --- |
| Female | 421 | 28.3 | 1031 | 36.8 |
| **Race**  White | 768 | 51.5 | 1254 | 44.7 |
| Black | 193 | 13.0 | 363 | 12.9 |
| Other | 529 | 35.5 | 1188 | 42.4 |
| **Age**  <65 | 583 | 39.1 | 398 | 14.2 |
| ≥65 | 907 | 60.9 | 2407 | 85.8 |
| **Stage**  Distant | 434 | 29.1 | 386 | 13.7 |
| Localized | 173 | 11.6 | 911 | 32.4 |
| Regional | 695 | 46.7 | 625 | 22.2 |
| Unknown | 188 | 12.6 | 883 | 31.4 |
| **Chemotherapy**  Yes | 1490 | 100 | / | / |
| No/Unknown | / | / | 2805 | 100 |
| **Radiotherapy**  Yes | 714 | 47.9 | 154 | 5.5 |
| Refuse | 5 | 0.3 | 37 | 1.3 |
| Unknown | 771 | 51.8 | 2614 | 93.2 |

Other: race including Asian or Pacific Islander and American Indian/Alaska Native.
